# DNA N^6^-methyladenine modifications of *Acidithiobacillus ferrooxidans* response to copper stress

**DOI:** 10.1371/journal.pone.0337584

**Published:** 2025-12-01

**Authors:** JingQi Liu, HuangFeng Qiu, DongHua Tan, Yu Zhang, Yu Yang

**Affiliations:** 1 School of Minerals Processing and Bioengineering, Central South University, Changsha, Hunan, China; 2 Xiangya School of Medicine, Central South University, Changsha, Hunan, China; 3 School of Minerals Processing and Bioengineering, Key Laboratory of Biohydrometallurgy of Ministry of Education, Central South University, Changsha, Hunan, China; Ruhr-Universitat Bochum, GERMANY

## Abstract

High concentrations of copper ions have long been recognized as a key factor limiting the efficiency of bioleaching due to the metal toxicity to microorganisms. In order to identify new determinants of copper resistance, we assessed the impact of different copper ion concentrations on the bioleaching model organism *Acidithiobacillus ferrooxidans*. Furthermore, we employed 6mA IP-seq technique to evaluate changes in the 6mA methylation levels of *A. ferrooxidans* under two conditions: iron oxidation and sulfur oxidation, both under copper stress. The results indicated that as the concentration of copper ions in the growth environment increased, the copper toxicity significantly inhibited the growth of *A. ferrooxidans*. The maximum tolerable copper ion concentration for iron-grown and sulfur-grown *A. ferrooxidans* was found to be 100 mM. Under 100 mM Cu^2+^ exposure, 184 and 242 differentially methylated genes were identified in the iron oxidation and sulfur oxidation *A. ferrooxidans*, respectively(P < 0.01). From the Kyoto Encyclopedia of Genes and Genomes (KEGG) functional analysis, under iron oxidation conditions, 130 differentially methylated genes were annotated and mapped into 7 KEGG pathways, while under sulfur oxidation conditions, 188 differentially methylated genes were annotated and mapped into 4 KEGG pathways (P < 0.05). Several differentially methylated genes were found to be associated with the following responses to copper stress: iron-sulfur oxidation acceleration, amino acid synthesis, and activation of the RND-type efflux system, polypeptide-based copper resistance systems, and metal ATPases to expel copper ions. In summary, the 6mA methylation levels in *A. ferrooxidans* change under copper stress, and these changes are widely present in various copper resistance genes. This study reveals a novel copper resistance mechanism in *A. ferrooxidans*, providing new insights for enhancing bioleaching efficiency and demonstrating significant implications for advancing biometallurgy.

## 1 Introduction

In recent years, bioleaching, which is defined as the microbial-mediated conversion of an insoluble metal into a soluble form, has attracted considerable attention as a promising, sustainable, and cost-effective alternative to conventional methods for extracting metals from low-grade ores [1,2]. The bioleaching environment is inevitably confronted with high concentrations of heavy metal ions that can be toxic to microorganisms, constituting one of the most crucial factors constraining the efficiency of bioleaching [3]. Copper, the most prevalent heavy metal ion in bioleaching environment, is an essential trace element in living organisms [4]. However, microorganisms employed in bioleaching are continuously exposed to bioleaching systems that contain high concentrations of copper, which most microorganisms cannot adapt to. When exposed to high copper environments such as heap leachates or dump leachates, where copper ion concentrations reach 6 g/L, and especially in bioleaching stirred-tank reactors, where levels can be as high as 19 g/L, intracellular copper accumulates to toxic concentrations [5]. This leads to disruption of bacterial copper homeostasis [6]. It has been observed that some bioleaching microorganisms exhibit a remarkable innate tolerance to elevated copper concentrations. [7]. As reported, *Acidithiobacillus ferrooxidans* (*A. ferrooxidans*) exhibits a tolerance threshold for copper ions reaching 800 mM, which substantially surpasses that of common bacterial species [8]. This phenomenon may be attributed to the evolutionary development of specific copper resistance strategies by these microorganisms during their long-term adaptation to such extreme environments [9]. Therefore, researchers have been searching for the copper resistance determinants in bioleaching microorganisms, aiming to provide new insights for enhancing bioleaching efficiency.

*A. ferrooxidans* is a Gram-negative, chemolithoautotrophic bacterium with an optimal growth pH of approximately 2. As the first strain isolated that can oxidize ferrous iron (Fe(Ⅱ)) and various reduced inorganic sulfides (RISCs) for energy, *A. ferrooxidans* has undergone extensive investigation and plays an important role in a variety of bioleaching systems [10]. Several genes linked to copper homeostasis in other bacteria have been identified in the genome of *A. ferrooxidans* ATCC 23270, and their presence is considered a contributing factor that enables the bacterium to survive under high copper concentrations. These include three genes coding for Cu^+^-transporting P-type ATPase (*copA1*_*Af*_, *copA2*_*Af*_, and *copB*_*Af*_), three genes coding for HME-RND family protein (CusCBA) associated with the release of copper ions from the cell (*cusA*_*Af*_, cusB_Af_, cusC_Af_), and two additional genes coding for periplasmic metal chaperone protein for the transport of this metal (*cusF*_*Af*_ and *copC*_*Af*_) [8,11]. In addition to the copper resistance genes, exposure to high concentrations of copper also results in changes in the expression levels of specific genes associated with iron-sulfur metabolism, tricarboxylic acid (TCA) cycle, histidine synthetase, and other crucial processes that are necessary for sustaining regular cellular growth in *A. ferrooxidans* [12,13]. These studies collectively indicate that *A. ferrooxidans* possesses a response mechanism to copper stress. While there is currently a comprehensive understanding of these mechanisms in recent years, the detailed mechanisms by which *A. ferrooxidans* regulates gene expression to mitigate cellular toxicity under copper stress remain unclear.

As essential subjects in the field of epigenetics research, DNA methylation can regulate gene expression by imparting different reversible regulatory states to the same gene sequence [14]. In prokaryotes, DNA methyltransferases(MTases) recognize specific gene target sequences and transfer the methyl group from S-adenosyl methionine (SAM) to these sequences, resulting in three forms of DNA methylation: N6-methyladenine (m6A), 5-methylcytosine (m5C), and N4-methylcytosine (m4C) [15]. Among these three DNA markers, 6mA is the most prevalent form in prokaryotes, which contrasts with the fact that 5mC is the predominant form in eukaryotes [16]. The DNA MTase can operate as part of the restriction-modification (R-M) system, working in collaboration with a restriction endonuclease (REase) to defend against the invasion of exogenous DNA, or function as an “orphan” MTase in the absence of a cognate restriction enzyme [17]. With the gradual investigation of methyltransferases in bacteria, it has been revealed that if the target sites of these two MTases are located within the promoter or regions associated with transcriptional regulation, both MTases can participate in transcriptional regulation [18]. In our previous study on the variation of genomic DNA methylation levels in *A. ferrooxidans* using two different culture substrates (Fe (II) and RISCs), we observed significantly higher levels of 6mA under Fe (II) oxidizing conditions compared to RISCs oxidizing conditions [19]. This observation suggests the involvement of methyltransferases in regulating and transforming iron-sulfur metabolism. Considering the alterations in the expression levels of iron-sulfur oxidation-related genes in *A. ferrooxidans* under copper stress, it is plausible that methylation regulation may also function as a strategy for coping with extreme environmental stress.

Currently, numerous studies have indicated that DNA methylation plays a crucial role in the response of higher eukaryotes to heavy metal ion stress. Under Cd stress, the expression levels of DNA methylation-related genes in rice significantly increase, and there are noticeable changes in the methylation levels of many genes related to stress response and metal transport [20]. When exposed to excess copper, *H. verticillata* can modulate its methylation levels in response to copper toxicity by either generating reactive oxygen species (ROS) to interfere with DNA methylation or by increasing the expression of proteins associated with DNA methylation [21]. Furthermore, significant increases in DNA methylation levels have been reported in earthworms, eels, and human embryo lung fibroblast cells after exposure to low doses of Cd [22–24]. However, there is limited research on whether DNA methylation in prokaryotes plays a similar role as in eukaryotes under heavy metal stress. A study on the alterations in DNA of heavy metal-acclimated *Gordonia sp*. reveals changes in global DNA methylation patterns in bacteria acclimated to Cd, Pb, and Ag. In contrast to the hypomethylation observed in Pb- and Ag-acclimated bacteria, Cd-acclimated bacteria exhibited hypermethylation compared to the control [25].

Overall, the regulation of methylation in *A. ferrooxidans* may be similar to that in plants, playing a crucial role in cellular responses to metal stress. The aim of this study is to explore the relationship between 6mA methylation regulation in *A. ferrooxidans* and copper resistance mechanisms. And this research seeks to provide new insights into potential determinants of copper resistance and offers novel approaches to overcoming the limitations of bioleaching efficiency.

## 2 Materials and methods

### 2.1 Bacterial strains and growth conditions

The experimental strain was *A. ferrooxidans* ATCC 23270 and obtained from the Key Laboratory of Biometallurgy of the Ministry of Education, Central South University, Hunan, China. The *A. ferrooxidans* strains were inoculated into a basic 9K medium containing FeSO_4_•7H_2_O (44.7g/L) or S^0^(10 g/L) at pH = 2, and incubated at 30°C with shaking at 180 rpm.

The main constituents of 9K basic medium contain the following (g/L): Ca(NO_3_)_2_, 0.01; KCl, 0.1; K_2_HPO_4_, 0.5; MgSO_4_·7H_2_O, 0.5; (NH_4_)_2_SO_4_, 3.0; The FeSO₄•7H₂O solution was sterilized by filtration, while elemental sulfur required autoclaving at 100°C under atmospheric pressure for 1 hour, performed three times with 24-hour intervals between each session.

### 2.2 Copper stress treatment

To investigate the mechanisms underlying copper ion stress on the growth of *A. ferrooxidans* under iron and sulfur oxidation conditions, the growth conditions of *A. ferrooxidans* ATCC 23270 were divided into two groups: iron oxidation and sulfur oxidation. This differentiation was achieved by using either FeSO_4_•7H_2_O or S^0^ as the sole nutritional substrate. Each group was subjected to six different concentrations of Cu^2+^ (0, 10, 50, 100, 150, 200 M) to assess the growth curves and maximum copper tolerance of *A. ferrooxidans*. Due to the slow growth of *A. ferrooxidans* under S^0^ oxidation conditions, there is no significant change in biomass within 12 hours. Therefore, the biomass of the Fe^2+^ oxidation group and the S^0^ oxidation group was measured at 12-hour and 24-hour intervals, respectively, using the hemocytometer counting method [26]. The specific growth rate μ of *A. ferrooxidans* under different cultivation conditions was calculated using the following formula [27]:


$$\mu =\frac{ln\;x_{\text{2}}-ln\;x_{\text{1}}}{t_{\text{2}}-t_{\text{1}}}$$

X_1_ and X_2_ represent the biomass at the start time t_1_ and end time t_2_ of the logarithmic growth phase, respectively.

### 2.3 Determination of ferrous iron

Every 12 hours, liquid samples were collected from iron-oxidizing *A. ferrooxidans* fermentation broth at various concentrations to determine the Fe^2+^ concentration. After centrifugation of the samples at 7500 g for 10 minutes, the supernatant was carefully collected and diluted as appropriate. Following the method described in standard methods [28], 0.5% (w/v) 1,10-phenanthroline solution and buffer solution (400 g/L CH₃COONH₄, 50% acetic acid, pH 3.2) were added to the diluted samples. After allowing the reaction to proceed at room temperature for a specified period, the optical density (OD) at 510 nm was measured using a UV-Vis spectrophotometer (UV-2550, Shimadzu, Japan) to calculate the concentration of Fe^2+^.

### 2.4 6mA IP-Seq and bioinformatic analyses

Genomic DNA extracted from *A. ferrooxidans* of each group was incubated with RNase overnight and then diluted to 100–200 ng/μL with TE buffer. Subsequently, the DNA was fragmented into 200−400 bp using an ultrasonic processor with the following parameters: 40 W and 50 kHz, sonication for 3 seconds, followed by a 5-second interval, with this process repeated for a total of 8 minutes. After end repair and adapter ligation of the fragmented DNA, the repaired DNA was denatured at 95°C for 10 minutes. A portion of the denatured DNA was retained as input, while the remaining portion was subjected to immunoprecipitation with 6mA antibody at 4°C overnight. The precipitated DNA was purified using proteinase K and then eluted by 6mA salt competition at 4°C. The eluate was frozen overnight at −80°C and then centrifuged at 14,000 g for 20 minutes. The DNA pellet was dissolved in ddH_2_O.

The immunoprecipitated DNA and input DNA were PCR amplified to construct sequencing libraries. Sequencing was performed on the Illumina NovaSeq 6000 platform (Illumina, USA). Raw data were trimmed using Solexa Pipeline Software v 1.8 (Off-Line Base Caller Software, v 1.8) and assessed for quality using FastQC (v 0.11.7). Based on the quality assessment, low-quality reads and sequencing adapters were filtered out using Trimmomatic, and duplicate reads were removed with SAMtools (v 1.9). Clean reads were mapped to the reference genome of *A. ferrooxidans* ATCC 23270 in the database using BWA (Burrows-Wheeler Aligner v 0.7.17) software for subsequent analysis. Integrative Genomics Viewer (IGV v 2.13.2) was used to visualize the differences in 6mA methylation levels of the genome under different treatment conditions. Differentially methylated genes under copper stress were functionally classified by Kyoto Encyclopedia of Genes and Genomes (KEGG) (http://www.genome.jp/kegg/, accessed on 24 October 2024) pathway enrichment analyses.

### 2.5 Statistical analysis

All experiments were performed at least three times. All the data are expressed as the mean±standard deviation unless otherwise stated. The relevant statistical analysis was performed using SPSS 20.0 software. *p < 0.05, **p < 0.01, or ***p < 0.001 was considered to indicate statistical significance.

## 3 Results and discussion

### 3.1 Analysis of physiological characteristics of *A. ferrooxidans* under copper stress

**Fig 1:**
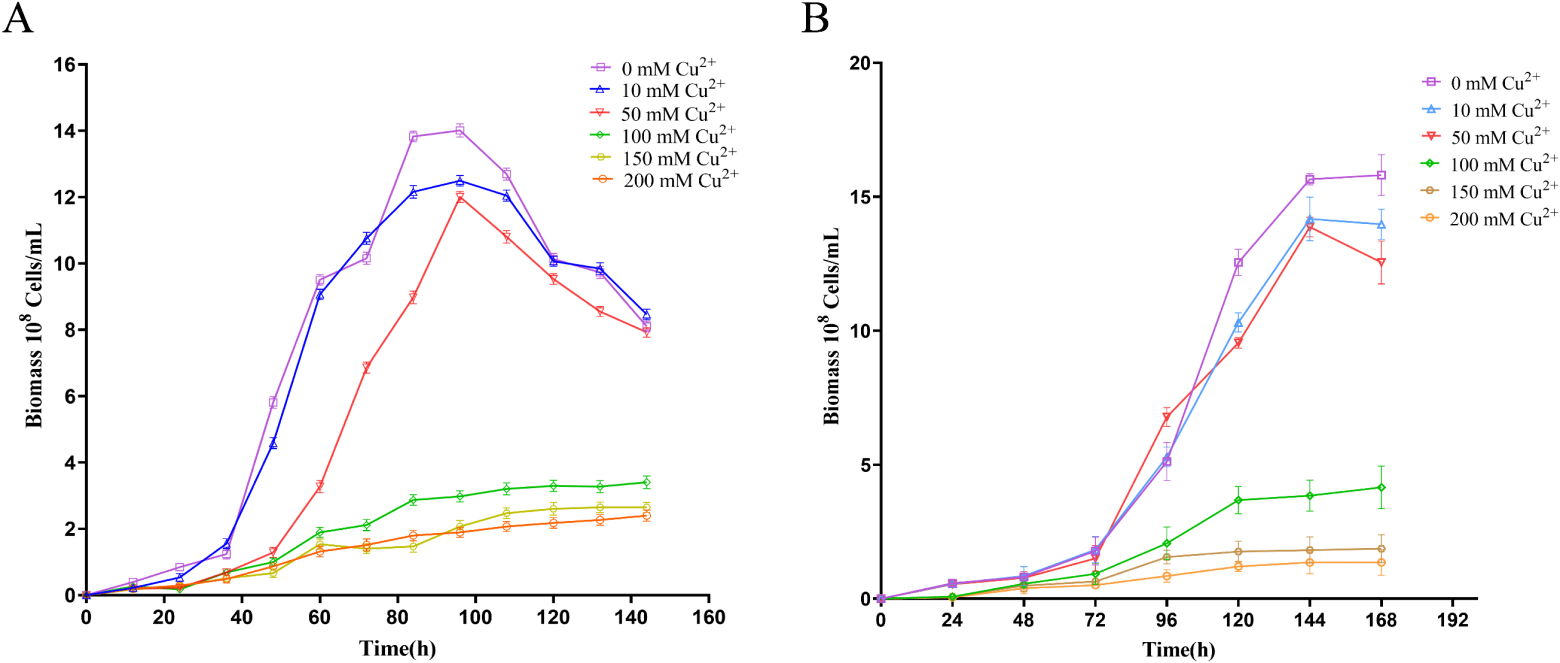
The growth curve of *A. ferrooxidans* under Fe²⁺ (A) and RISCs (B) oxidation conditions with the addition of different concentrations of Cu²⁺.

**Fig 2:**
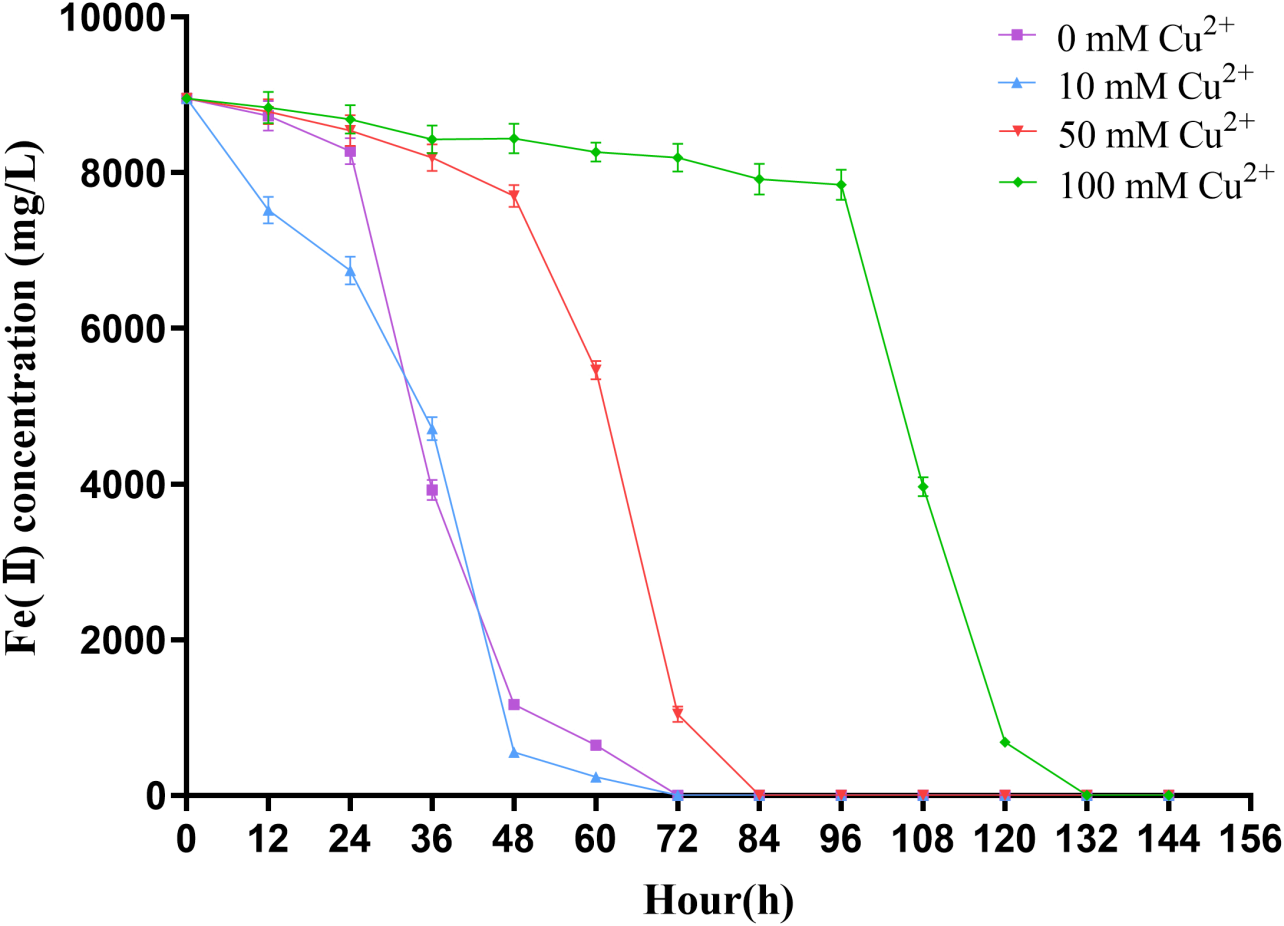
Dynamic changes of Fe^2^^+^ concentration in *A. ferrooxidans* culture under different copper ion stress levels.

As illustrated in Fig 1A, the growth characteristics of *A. ferrooxidans* ATCC 23270 under the condition of Fe^2+^ oxidation exhibited significant changes under varying levels of copper stress. Compared to the groups with 50 mM and 100 mM Cu2+, which showed a distinct lag phase at 0-24h, the group with copper stress levels less than 10 mM entered the logarithmic phase directly at 12-96h, without displaying a lag phase. In addition, when the copper concentration exceeded 150 mM, the growth curve of *A. ferrooxidans* flattened out, showing no apparent logarithmic phase. This indicated that copper concentrations of 150 mM and 200 mM exceeded the maximum tolerance level of *A. ferrooxidans* for copper stress, leading to severe growth inhibition. Additionally, the biomass of *A. ferrooxidans* ATCC 23270 decreased progressively with increasing copper stress, particularly at a concentration of 100 mM Cu2+, where the biomass amounted to only 2.6 × 10^8^ cells/mL, representing a significant reduction of 79.2% compared to the control group. To further investigate the impact of copper on the growth of *A. ferrooxidans*, we measured the maximum specific growth rate ($\mu_{max}$) and Fe^2+^ oxidation rate of *A. ferrooxidans* exposed to copper concentrations ranging from 0–100 mM. The results revealed maximum growth rates ($\mu_{max}$) of 0.12 h^-1^, 0.10 h^-1^, 0.08 h^-1^, and 0.06 h^-1^ for the groups with 0 mM, 10 mM, 50 mM, and 100 mM Cu2+, respectively. Fig 2 shows the variation in Fe2+ concentration in the solution as a function of growth time. As the concentration of copper increased, the time required for complete oxidation of Fe^2+^ in the solution also increased. Fe2+ was completely oxidized within 72 hours when the copper concentration in the solution was below 10 mM. However, when the copper concentration increased to 100 mM, the time required for complete oxidation of Fe2+ extended to 132 hours. The expression levels of Fe oxidation-related genes in *A. ferrooxidans* decreased in the presence of copper, which adversely affected respiration [29]. This not only led to an extension of the time required for complete oxidation of Fe^2+^ in the culture but also contributed to the prolonged growth cycle and reduced biomass of *A. ferrooxidans*.

Under S^0^ oxidation, groups with 10 mM and 50 mM Cu2+ entered the logarithmic phase directly at 48-144h, similar to the control group. In contrast, the groups with 100 mM Cu2+ exhibited a lag phase at 0-24h, comparable to that observed under Fe^2+^ oxidation conditions. When copper stress exceeded 150 mM, the growth of *A. ferrooxidans* in S^0^ was completely inhibited. Consistent with observations under Fe^2+^ oxidation conditions, the biomass of *A. ferrooxidans* was inversely proportional to the copper stress levels. As shown in Fig 1B, compared to the biomass of *A. ferrooxidans* without copper stress, which was 1.6 × 10⁹ cells/mL, the 10 mM and 50 mM Cu2+ treatments caused only slight reductions in biomass, while the 100 mM group showed a significant decrease to 4.15 × 10⁸ cells/mL, representing a 74.1% decline. Similarly, we measured the specific growth rates of *A. ferrooxidans* under S^0^ oxidation conditions at copper concentrations of 0, 10, 50, and 100 mM. The results revealed $\mu_{max}$values of 0.06 h^-1^, 0.05 h^-1^, 0.05 h^-1^, and 0.04 h^-1^, respectively. The hydrophobic nature of the S monomer affects cell’s utilization of sulfur [30], which may explain why *A. ferrooxidans* under S^0^ oxidation conditions experience a longer growth cycle and lower $\mu_{max}$ under the same copper stress compared to Fe^2+^ oxidation conditions.

Based on the above considerations, irrespective of the growth substrate (Fe^2+^ or S^0^), the inhibition of *A. ferrooxidans* growth increases progressively with the rising levels of copper stress. Moreover, 100 mM copper concentration represents the maximum tolerance level for *A. ferrooxidans*. Therefore, *A. ferrooxidans* ATCC 23270 exposed to 100 mM copper was selected as a suitable experimental subject for extracting the genome to investigate changes in 6mA levels.

### 3.2 Variation of N^6^-methyladenine modification due to copper exposure

**Fig 3:**
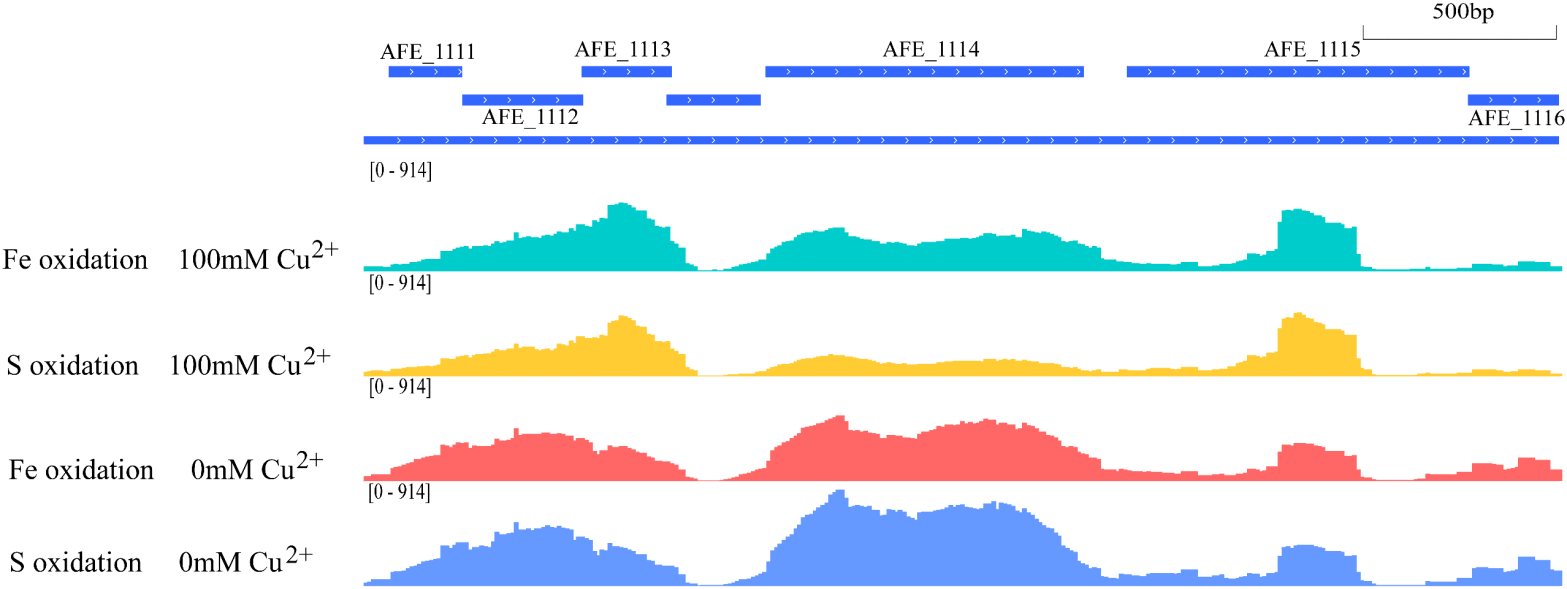
Distribution of 6mA in selected genes. Snapshot of 6mA sites in selected genes. The data is derived from 6mA-IP-seq experiments conducted on A. *ferrooxidans* under various growth conditions. Genome annotations are shown on the top line.

We employed the 6mA IP-Seq technique to identify differentially methylated genes in *A. ferrooxidans* ATCC 23270 subjected to 100 mM Cu^2+^ under both iron oxidation and sulfur oxidation conditions. The results indicated that a total of 184 differentially methylated genes were identified under iron oxidation, while 242 differentially methylated genes were identified under sulfur oxidation (P < 0.01). As illustrated in Fig 3, the changes in 6mA methylation levels of specific genes under copper stress are presented more intuitively. Previous experiments demonstrate that copper stress negatively impacts *A. ferrooxidans*. The observed alterations in gene methylation levels suggest that 6mA methylation may be involved in regulating the expression of copper stress-related genes.

#### 3.2.1 KEGG annotated and classified the differentially methylated genes.

**Fig 4:**
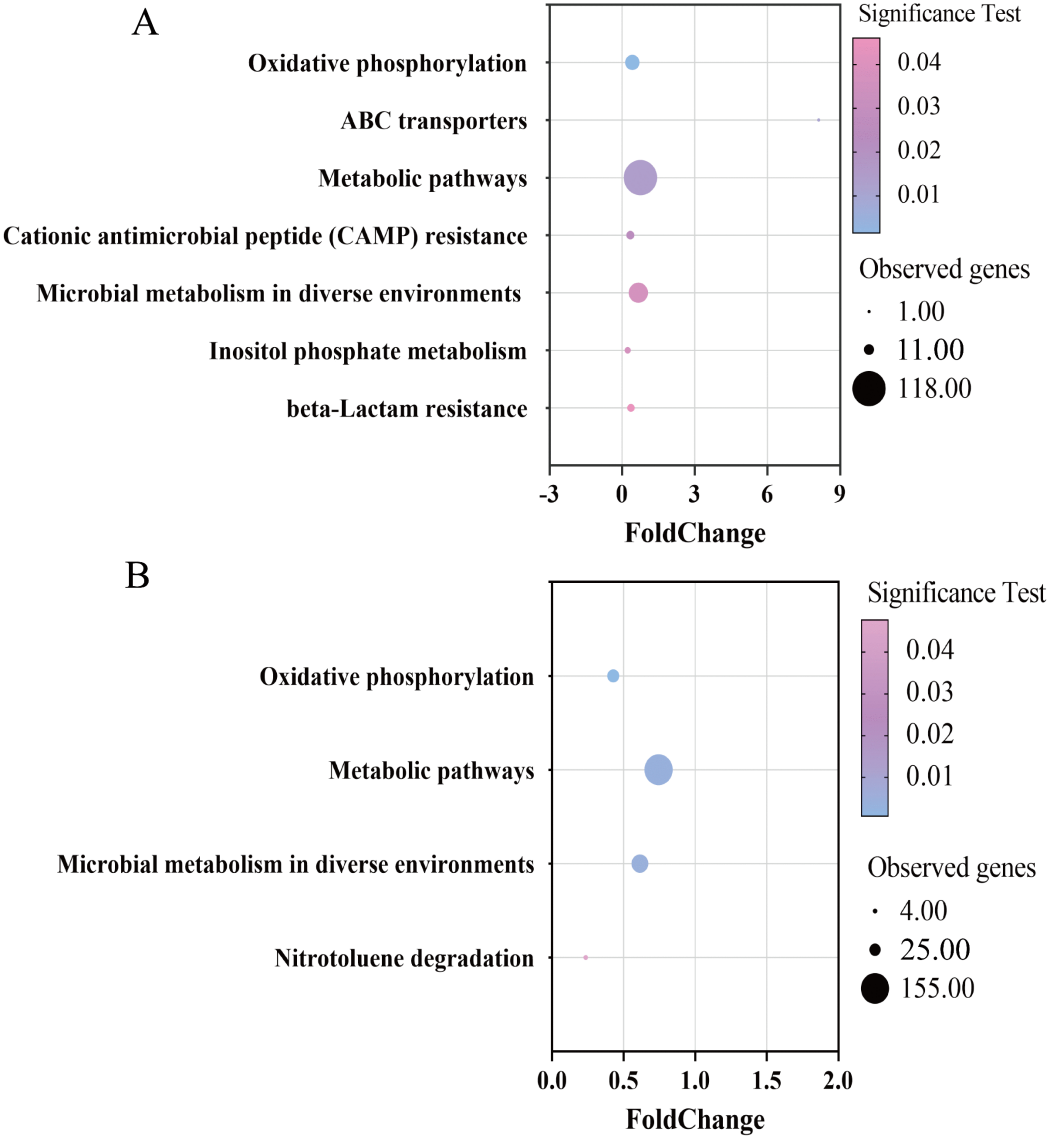
KEGG functional enrichment analyses of the differentially methylated genes for iron oxidation (A) and sulfur oxidation (B) *A. ferrooxidans* cultivated under copper stress The fold change represents the degree of enrichment of the differentially methylated genes. The Y-axis shows the names of the enriched pathways. The area of each node represents the number of observed genes. The significance test is represented by a color scale.

The KEGG classification of differentially methylated genes under copper stress was employed to infer the role of 6mA in assisting cells in coping with copper stress. From the KEGG functional analysis, under iron oxidation conditions, 130 differentially methylated genes were annotated and mapped into 7 KEGG pathways, while under sulfur oxidation conditions, 188 differentially methylated genes were annotated and mapped into 4 KEGG pathways (P < 0.05). Furthermore, some differentially methylated genes may be involved in multiple KEGG pathways. The differentially methylated genes annotated under iron oxidation and sulfur oxidation conditions are listed in Supplementary S1 and S2 Tables, respectively. Three main pathways were found to be enriched for the differentially methylated genes under iron oxidation: metabolic pathways, oxidative phosphorylation and microbial metabolism in diverse environments (Fig 4B). For the differentially methylated genes under sulfur oxidation, the most enriched pathways were oxidative phosphorylation and metabolic pathways (Fig 4C). The following section analyzes these differentially methylated genes in conjunction with their functional annotations.

#### 3.2.2 Oxidative phosphorylation.

In *A. ferrooxidans* under copper stress, the methylation levels of several genes (*nuoH, nuoI, nuoJ, nuoK, nuoM, nuoN*) encoding NADH dehydrogenase complex involved in the NADH dehydrogenase pathway and those genes (*coxA, coxB, coxC*) encoding aa3-type cytochrome oxidase complex involved in the cytochrome c oxidase pathway decreased. When *A. ferrooxidans* utilizes iron as an energy substrate, electrons obtained from iron flow through the c-type cytochrome Cyc2 to the periplasmic copper protein rusticyanin. A portion of these electrons is transferred through cytochrome c4 (Cyc1) and the aa3-type cytochrome oxidase complex to reduce oxygen to water, constituting a “downhill electron pathway”. And another portion of the electrons is sequentially transferred through cytochrome c4 (CycA1), the bc1 complex encoded by the *petI* operon, and membrane-associated quinones, ultimately providing reducing power to NADH dehydrogenase I. This pathway is referred to as the “uphill electron pathway” [31]. In *A. ferrooxidans* ATCC 53993 growing under copper stress, the protein levels of two subunits of the cytochrome oxidase aa3 (CoxA and CoxB) increased, likely due to the need for more of these proteins as iron-containing prosthetic groups of heme-containing proteins to counteract copper-induced iron deficiency in the cytoplasm [32]. Furthermore, the expression levels of these two proteins also increased in *A. ferrooxidans* ATCC 23270 under sulfur cultivation conditions when subjected to copper stress [33].

Compared to the Fe^2+^ oxidation pathway, the RISCs oxidation pathway in *A. ferrooxidans* is more complex. Electrons generated from the reduction of elemental sulfur enter the inner membrane via Heterodisulfide Reductase (Hdr)-like proteins and are subsequently transmitted to quinol pool. From this point, some electrons are transferred to NADH dehydrogenase complex to generate NADH, while others can be directed to terminal oxidases bd or bo3 for the reduction of oxygen to water. Additionally, the bc1 complex, encoded by the *petⅡ* operon, can also obtain electrons from quinol pool and transfer them to terminal oxidases via either cytochrome c or high potential iron–sulfur proteins (HiPIPs) for the reduction of oxygen [34,35]. According to the results, under copper stress, the methylation levels of genes encoding the terminal oxidase bo3 (*cyoA, cyoB, cyoC, cyoD*) and the terminal oxidase bd (*cydA),* as well as certain genes (*petB-2, petC-2*) of the bc1 complex encoded by the *petII* operon, decreased in both iron and sulfur oxidation *A. ferrooxidans*.

The methylation levels of the *atpA*, *atpD*, *atpG*, and *atpH* genes, which encode subunits of the F-type H^+^ transfer ATP synthase, decreased in sulfur oxidation cells under copper stress. Under aerobic conditions, the majority of ATP in bacteria is produced by the F-type ATP synthase through a transmembrane proton flow driven mechanism. Under copper stress, cells often require higher levels of ATP to activate copper efflux systems such as Cu transport ATPase CopA1, CopA2, CopB [36]. The observed changes in the methylation levels of the aforementioned genes may relate to altered ATP demands under copper stress.

The polyphosphate-based copper resistance system, composed of polyphosphate synthase (PPK) and polyphosphate kinase (PPX), which is responsible for the hydrolysis of polyphosphate to release inorganic phosphate (Pi), serves as a crucial copper-resistance determinant. Polyphosphate (polyp) is synthesized from ATP through the action of PPK, and in the presence of copper, PPX degrades polyP, forming copper ion-phosphate complexes that are expelled from the cell via the Pi carrier system or the phosphate transporter Pho84 [37]. In *A. ferrooxidans* under copper stress, the methylation levels of the gene encoding polyphosphate synthase (*ppk1*) also exhibited a decline.

#### 3.2.3 Metabolic pathways.

In the presence of high concentrations of copper, the methylation levels of genes associated with cysteine synthesis (*cysE*) in the genome of *A. ferrooxidans* decreased in both iron and sulfur culture media. In contrast, the histidine regulatory genes *(hisA*, *hisF* and *hisIE*) exhibited low methylation level only under sulfur oxidation conditions. In the study by Rodrigo et al [38], on the transcriptional level changes of *A. ferrooxidans* exposed to copper-containing environments, an increase in the expression levels of these genes was observed. Both cysteine and histidine serve as amino acid substrates for the synthesis of key antioxidant, such as glutathione, and both show a strong affinity for divalent metals, thereby playing crucial roles in protecting cells from heavy metal toxicity [39,40]. In *Acidithiobacillus caldus* that underwent long-term copper tolerance acclimatization, cells secreted increased levels of glutamic acid, glycine, and cysteine to facilitate the synthesis of glutathione, thereby reducing peroxide damage caused by copper stress [41]. After knocking out the histidine synthesis-related gene *HisB* in *Aspergillus fumigatus*, the mutant strain lacking *HisB* was unable to grow in media containing high concentrations of copper, cobalt, and nickel, where the wild-type strain can thrive. However, upon the exogenous addition of histidine, the mutant strain exhibited restored resistance to the three heavy metals [42]. In previous study, under copper stress, the expression levels of genes involved in cysteine synthesis were upregulated in the metal-resistant bacterium *Cupriavidus gilardii* [43]. The upregulation of both cysteine and histidine synthesis pathways may be associated with the direct interaction of these amino acids with copper ions to mitigate copper toxicity, or it may reflect an increased demand for these amino acids due to their involvement in the synthesis of copper detoxification-related proteins. Moreover, in sulfur oxidation cells under copper stress, the methylation levels of the glutamine synthetase gene *glnA*, which is involved in glutamic acid metabolism, and the gene *serB*, which is associated with serine synthesis, decreased. In contrast, in iron oxidation cells, in addition to the aforementioned genes, the gene argH related to arginine synthesis also exhibited low methylation levels. Amino acids play a crucial role in various metabolic pathways, and changes in amino acid metabolism have been observed under copper stress in several organisms, such as *A. ferrooxidans, Escherichia coli*, and *Saccharomyces cerevisiae*. Furthermore, alterations in the methylation levels of genes associated with amino acid metabolism may suggest that 6mA methylation is involved in the regulatory processes of genes related to amino acid metabolism in response to copper ion stress.

When excessive Cu^2+^ is present in the cell, the RND-type efflux system is primarily employed for the release of Cu^2+^ due to its energy efficiency. The RND-type efflux system typically consists of three components: an inner membrane pump, a periplasmic fusion protein, and an outer membrane channel protein. In *A. ferrooxidans*, the CusABC efflux system, which is widely studied for its role in Cu^2+^ release, utilizes the proton gradient across the membrane to expel Cu^2+^ from the cell while protons enter the cell [44]. As reported by Rodrigo et al, under stress conditions with 50 mM Cu^2+^, the expression of CusABC is significantly upregulated in both iron and sulfur oxidation *A. ferrooxidans* [45]. In the presence of 100 mM Cu^2+^, a downregulation of the methylation levels of genes *AFE_0110* and *AFE_1878*, which encode membrane transport proteins, was observed in iron-oxidizing *A. ferrooxidans*. However, there was no such phenomenon detected in sulfur oxidation cells. Based on the genomic context of these two genes, there are genes adjacent to them that are responsible for encoding inner membrane pumps and peribacteroid membrane fusion proteins.The genetic background of these genes is similar to that of the gene cluster *cusCBA*, which encodes the RND-type efflux system in *A. ferrooxidans*. Therefore, we hypothesize that *AFE_0110* and *AFE_1878* may belong to two novel RND-type efflux systems. According to Rodrigo et al [45], in the presence of copper, the expression of protein AFE_1878 is upregulated in iron oxidation *A. ferrooxidans*. In previous reviews, it is evident that the RND-type efflux system plays a crucial role in metal resistance [46]. Thus, the observed changes in methylation indicate a potential regulation relationship between the two systems.

**Fig 5:**
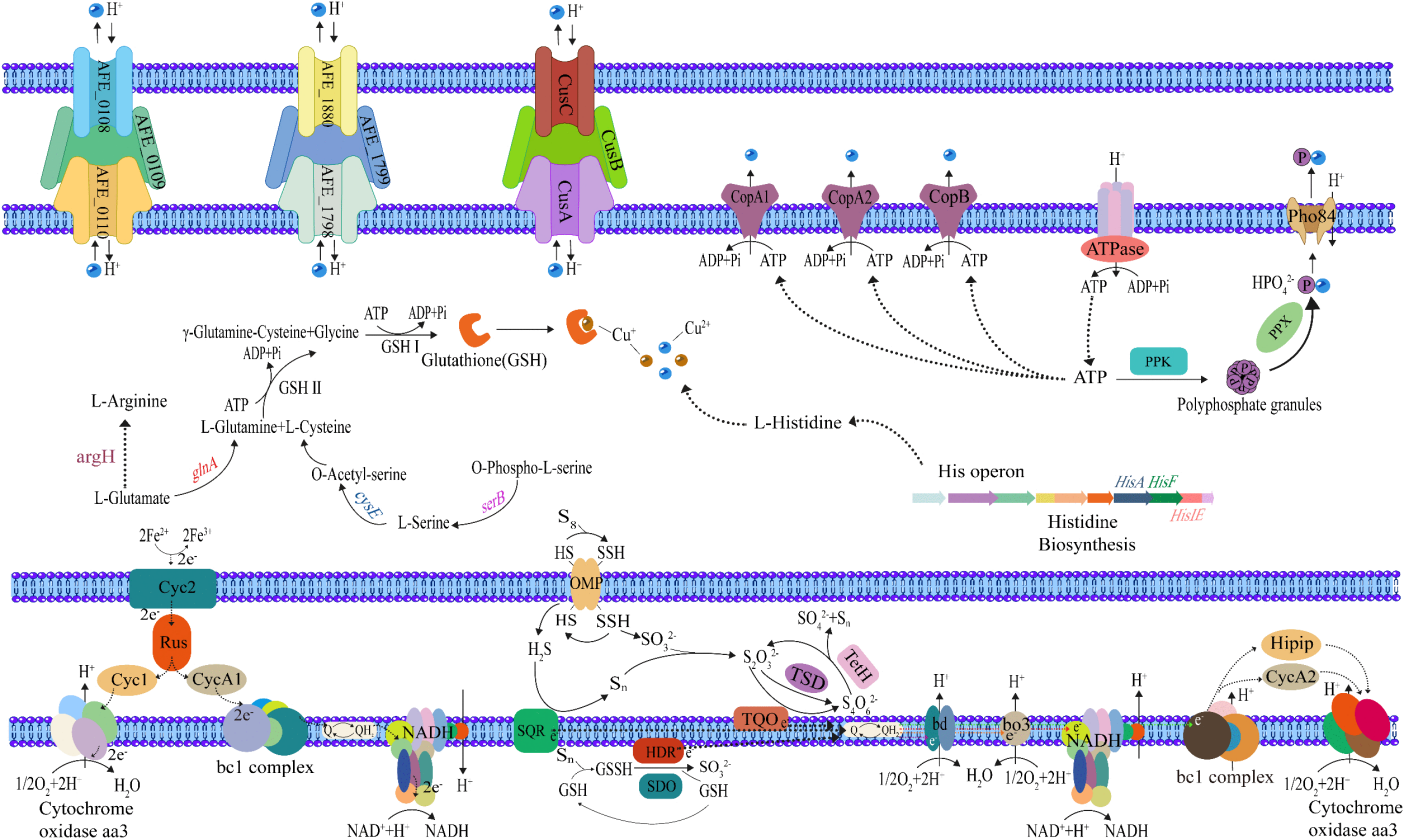
The copper resistance mechanism associated with differentially methylated genes in *A. ferrooxidans* ATCC 23270.

Currently, research on the epigenetic regulation under metal stress has primarily focused on the plant domain. When rice grows in the presence of Cd, the methylation level of the gene encoding the plasma membrane transport protein *OsZIP1* decreasesd, an event believed to be associated with the upregulation of OsZIP1 [47]. Additionally, in wheat subjected to Cd, Pb, and Zn stress, the methylation levels of genes encoding transporters responsible for metal vacuolar sequestration *TaABCCs* decreased, accompanied by increased expression levels [48]. The alterations in methylation levels of these genes, which play a crucial role in metal stress response and tolerance, are thought to be associated with the regulation of their expression to alleviate metal toxicity under metal stress [49]. In the present study, we observed changes in the 6mA methylation levels of several genes encoding copper-resistance determinants in *A. ferrooxidans* under copper stress, which parallels observations made in plants. Therefore, we hypothesize that 6mA modification similarly correlates with gene regulation in *A. ferrooxidans* and plays an important role in coping with copper stress. Fig 5 presents a model summarizing some of the copper stress response mechanisms associated with the differentially methylated genes observed in this study. These copper toxicity resistance mechanisms involve several aspects: the acceleration of iron-sulfur oxidation processes, which provide a primary energy source to maintain cellular copper homeostasis. The activities of various enzymes related to amino acid synthesis, as well as ATP synthesis-related enzymes, are also altered. Moreover, the activation of RND-type efflux systems, polypeptide-based copper resistance systems, and metal-exporting ATPases facilitates the extrusion of copper ions, thereby alleviating cellular copper toxicity. These findings not only provide a novel direction for research into the copper resistance mechanisms of *A. ferrooxidans*, but also broaden our understanding of the regulatory functions of 6mA methylation in bacterium. Furthermore, with the transition of culture substrates, the genes exhibiting altered 6mA levels also vary. This phenomenon, wherein differences in methylation levels are observed between iron-oxidizing and sulfur-oxidizing cells under the same conditions, has also been documented in our previous study on the variations in the degree of genomic DNA 6-methyladenine modifications in *A. ferrooxidans* with two different culture substrates. This suggests that 6mA regulation is more complex in *A. ferrooxidans*.

## 4 Conclusion

This study investigated the changes in the 6mA methylation levels of *A. ferrooxidans* under copper exposure during both iron oxidation and sulfur oxidation condition. The results indicated that while methylation levels are altered under copper stress for both growth conditions, with most differentially methylated genes exhibiting hypomethylation, the specific differentially methylated genes identified in iron oxidation and sulfur oxidation are not entirely the same. *A. ferrooxidans* modulates the expression of differentially methylated genes, thereby enhancing the iron-sulfur oxidation process, regulating the activities of amino acid synthases, ATP synthesis-related enzymes, and metal-exporting ATPases, and activating the RND-type efflux systems along with the polypeptide-based copper resistance systems. Ultimately, these mechanisms collectively alleviate cellular copper toxicity.In summary, there is a correlation between 6mA methylation regulation and copper resistance mechanisms. Although the exact role of methylation in these copper resistance mechanisms remains to be further explored, this finding undoubtedly offers new insights for identifying novel copper resistance factors.

## Supporting information

S1 Table Methylated differentially expressed genes in enrichment pathways under iron oxidation The symbol “+” indicates an increase in gene methylation levels under copper stress, while the symbol “-” denotes a decrease in gene methylation levels. (PDF)

S2 Table Methylated differentially expressed genes in enrichment pathways under sulfur oxidation The symbol “+” indicates an increase in gene methylation levels under copper stress, while the symbol “-” denotes a decrease in gene methylation levels. (PDF)
