# Supplementary material for: DNA N6-methyladenine modifications of Acidithiobacillus ferrooxidans response to copper stress
Source: PLoS One. 2025 Dec 1;20(12):e0337584. doi: 10.1371/journal.pone.0337584 (PMC12668529; doi:10.1371/journal.pone.0337584)
Supplement: S2 Table — (PDF) [file pone.0337584.s002.pdf]

Table S2 Methylated differentially expressed genes in enrichment pathways under sulfur oxidation

Term Name	GeneID	Description	Degree of gene methylation
**Oxidative phosphory-lation**	*cyoA*	ubiquinol oxidase subunit II	-
	cyoB	cbb3-type cytochrome c oxidase subunit I	-
	cyoC	cytochrome c oxidase subunit 3	-
	cyoD	cytochrome o ubiquinol oxidase	-
	cyoE-1	heme o synthase	-
	cydA	cytochrome ubiquinol oxidase subunit I	-
	nuoN	NADH-quinone oxidoreductase subunit NuoN	-
	nuoM	NADH-quinone oxidoreductase subunit M	-
	nuoL	NADH-quinone oxidoreductase subunit L	-
	nuoK	NADH-quinone oxidoreductase subunit NuoK	-
	nuoJ	NADH-quinone oxidoreductase subunit J	-
	nuoI	NADH-quinone oxidoreductase subunit NuoI	-
	nuoH	NADH-quinone oxidoreductase subunit NuoH	-
	nuoB	NADH-quinone oxidoreductase subunit B	-
	nuoA	NADH-quinone oxidoreductase subunit A	-
	petB-2	cytochrome bc complex cytochrome b subunit	-
	petC-2	cytochrome c1	-
	coxA	hypothetical protein	-
	coxC	cbb3-type cytochrome c oxidase subunit I	-
	coxB	cytochrome c oxidase subunit II	-
	ppK1	polyphosphate kinase 1	-
	atpD	F0F1 ATP synthase subunit beta	-
	atpG	F0F1 ATP synthase subunit gamma	-
	atpA	F0F1 ATP synthase subunit alpha	-
	atpH	F0F1 ATP synthase subunit delta	-
	AFE_0098	FAD-dependent oxidoreductase	-
**Metabolic pathways**	*hisA*	1-(5-phosphoribosyl)-5-[(5-phosphoribosylamino)methylideneamino]imidazole-4-carboxamide isomerase	-
	hisF	imidazole glycerol phosphate synthase subunit HisF	-
	hisIE	bifunctional phosphoribosyl-AMP cyclohydrolase/phosphoribosyl-ATP diphosphatase HisIE	-
	serB	phosphoserine phosphatase SerB	-
	glnA	glutamate--ammonia ligase	-
	cysE	serine O-acetyltransferase	-
	AFE_0915	acetyl-CoA carboxylase biotin carboxylase	-

	subunit	
AFE_0068	7-cyano-7-deazaguanine synthase QueC	-
AFE_0069	7-carboxy-7-deazaguanine synthase QueE	-
AFE_0138	lipopolysaccharide heptosyltransferase II	-
AFE_0179	ferrochelatase	-
AFE_0203	D-alanine--D-alanine ligase	-
AFE_0232	O-antigen ligase family protein	-
AFE_0235	lipopolysaccharide heptosyltransferase I	-
AFE_0243	8-amino-7-oxononanoate synthase	-
AFE_0288	5-methyltetrahydropteroyltriglutamate--homocysteine S-methyltransferase	-
AFE_0289	bifunctional demethylmenaquinone methyltransferase/2-methoxy-6-polyprenyl-1,4-benzoquinol methylase UbiE	-
AFE_0293	homoserine O-acetyltransferase	-
AFE_0297	bifunctional diaminohydroxyphosphoribosylaminopyrimidine deaminase/5-amino-6-(5-phosphoribosylamino)uracil reductase RibD	-
AFE_0298	riboflavin synthase	-
AFE_0300	6,7-dimethyl-8-ribityllumazine synthase	-
AFE_0320	DNA-directed RNA polymerase subunit beta	-
AFE_0321	DNA-directed RNA polymerase subunit beta'	-
AFE_0423	aconitate hydratase	-
AFE_0527	glycogen/starch/alpha-glucan phosphorylase	-
AFE_0539	sulfate adenylyltransferase	-
AFE_0540	phosphoribosyltransferase family protein	-
AFE_0570	protoporphyrinogen oxidase	-
AFE_0592	4-hydroxybenzoate octaprenyltransferase	-
AFE_0593	chorismate lyase	-
AFE_0628	pyridoxamine 5'-phosphate oxidase	-
AFE_0629	ribose-5-phosphate isomerase RpiA	-
AFE_0631	ubiquinol oxidase subunit II	-
AFE_0632	cbb3-type cytochrome c oxidase subunit I	-
AFE_0633	cytochrome c oxidase subunit 3	-
AFE_0634	cytochrome o ubiquinol oxidase	-
AFE_0635	heme o synthase	-
AFE_0660	malic enzyme-like NAD(P)-binding protein	-
AFE_0692	FdhF/YdeP family oxidoreductase	-
AFE_0729	glutamate synthase small subunit	-
AFE_0730	glutamate synthase large subunit	-
AFE_0733	3-dehydroquinate synthase	-
AFE_0734	shikimate kinase	-
AFE_0745	biosynthetic-type acetolactate synthase large	-

	subunit	
AFE_0746	acetolactate synthase small subunit	-
AFE_0747	ketol-acid reductoisomerase	-
AFE_0748	phosphatidylserine decarboxylase	-
AFE_0749	pyruvate kinase	-
AFE_0750	CDP-diacylglycerol--serine O-phosphatidyltransferase	-
AFE_0778	ureidoglycolate lyase	-
AFE_0779	ring-opening amidohydrolase	-
AFE_0893	S-methyl-5-thioribose-1-phosphate isomerase	-
AFE_0900	prephenate dehydrogenase/arogenate dehydrogenase family protein	-
AFE_0901	3-phosphoshikimate 1-carboxyvinyltransferase	-
AFE_0902	(d)CMP kinase	-
AFE_0907	orotidine-5'-phosphate decarboxylase	-
AFE_0908	orotate phosphoribosyltransferase	-
AFE_0955	cytochrome ubiquinol oxidase subunit I	-
AFE_0958	NAD(P)/FAD-dependent oxidoreductase	-
AFE_1403	pyridoxine 5'-phosphate synthas	-
AFE_1405	UDP-glucose/GDP-mannose dehydrogenase family protein	-
AFE_1455	3-hydroxyacyl-ACP dehydratase FabZ	-
AFE_1456	acyl-ACP--UDP-N-acetylglucosamine O-acyltransferase	-
AFE_1471	biosynthetic arginine decarboxylase	-
AFE_1507	cysteine desulfurase NifS	-
AFE_1521	nitrogenase molybdenum-iron protein alpha chain	-
AFE_1522	nitrogenase iron protein	-
AFE_1537	aminotransferase class V-fold PLP-dependent enzyme	-
AFE_1663	glycolate oxidase subunit GlcF	-
AFE_1664	glycolate oxidase subunit GlcE	-
AFE_1665	FAD-linked oxidase C-terminal domain-containing protein	-
AFE_1778	glutamine-hydrolyzing GMP synthase	-
AFE_1799	alpha-glucan family phosphorylase	-
AFE_1800	enolase	-
AFE_1815	2,3-bisphosphoglycerate-independent phosphoglycerate mutase	-
AFE_1816	phosphoenolpyruvate synthase	-
AFE_1820	2,3,4,5-tetrahydropyridine-2,6-dicarboxylate N-succinyltransferase	-
AFE_1821	succinyldiaminopimelate transaminase	-

AFE_1873	aminopeptidase N	-
AFE_1893	dihydrolipoyl dehydrogenase	-
AFE_1897	phosphoribosylformylglycinamidine cyclo-ligase	-
AFE_1900	CDP-alcohol phosphatidyltransferase family protein	-
AFE_1908	3-oxoacyl-ACP reductase FabG	-
AFE_1910	beta-ketoacyl-ACP synthase II	-
AFE_1913	dTMP kinase	-
AFE_1928	tRNA (adenosine(37)-N6)-dimethylallyltransferase MiaA	-
AFE_1933	flavodoxin-dependent (E)-4-hydroxy-3-methylbut-2-enyl-diphosphate synthase	-
AFE_1962	c-type cytochrome	-
AFE_2064	amidophosphoribosyltransferase	-
AFE_2066	cyanophycin synthetase	-
AFE_2068	tryptophan synthase subunit alpha	-
AFE_2069	tryptophan synthase subunit beta	-
AFE_2081	malto-oligosyltrehalose synthase	-
AFE_2110	Asp-tRNA(Asn)/Glu-tRNA(Gln) amidotransferase subunit GatB	-
AFE_2131	aldehyde dehydrogenase family protein	-
AFE_2206	carboxylating nicotinate-nucleotide diphosphorylase	-
AFE_2222	glutamate--tRNA ligase	-
AFE_2223	PBP1A family penicillin-binding protein	-
AFE_2261	phosphoribosylamine--glycine ligase	-
AFE_2263	oxygen-dependent coproporphyrinogen oxidase	-
AFE_2288	phospholipase C, phosphocholine-specific	-
AFE_2289	hypothetical protein	-
AFE_2350	D-alanyl-D-alanine carboxypeptidase family protein	-
AFE_2543	radical SAM protein	-
AFE_2550	CoB--CoM heterodisulfide reductase iron-sulfur subunit B family protein	-
AFE_2551	4Fe-4S dicluster domain-containing protein	-
AFE_2553	FAD-dependent oxidoreductase	-
AFE_2554	heterodisulfide reductase-related iron-sulfur binding cluster	-
AFE_2633	triose-phosphate isomerase	-
AFE_2635	dihydropteroate synthase	-
AFE_2663	4-hydroxy-tetrahydrodipicolinate reductase	-
AFE_2730	cytochrome bc complex cytochrome b subunit	-
AFE_2731	cytochrome c1	-
AFE_2818	alanine racemase	-

	AFE_2825	beta-N-acetylhexosaminidase	-
	AFE_2923	D-alanyl-D-alanine carboxypeptidase family protein	-
	AFE_2924	glucose-6-phosphate isomerase	-
	AFE_3011	aminotransferase class I/II-fold pyridoxal phosphate-dependent enzyme	-
	AFE_3032	N-acetylmuramate alpha-1-phosphate uridylyltransferase MurU	-
	AFE_3051	form I ribulose bisphosphate carboxylase large subunit	-
	AFE_3052	ribulose bisphosphate carboxylase small subunit	-
	AFE_3122	NADPH-dependent assimilatory sulfite reductase hemoprotein subunit	-
	AFE_3123	phosphoadenylyl-sulfate reductase	-
	AFE_3124	sulfate adenylyltransferase subunit CysD	-
	AFE_3125	GTP-binding protein	-
	AFE_3138	2OG-Fe(II) oxygenase	-
	AFE_3148	hypothetical protein	-
	AFE_3149	cbb3-type cytochrome c oxidase subunit I	-
	AFE_3150	cytochrome c oxidase subunit II	-
	AFE_3239	2-polyprenyl-3-methyl-6-methoxy-1,4-benzoquinone monooxygenase	-
	AFE_3242	indole-3-glycerol phosphate synthase TrpC	-
	AFE_3249	pyruvate kinase	-
	AFE_3250	phosphoglycerate kinase	-
	AFE_3251	type I glyceraldehyde-3-phosphate dehydrogenase	-
	AFE_3252	transketolase	-
	AFE_3253	class II fructose-bisphosphatase	-
	AFE_3254	inositol monophosphatase family protein	-
	AFE_3300	hydroxymethylbilane synthase	-
	AFE_3303	glutamate-1-semialdehyde 2,1-aminomutase	-
	AFE_3304	thiamine phosphate synthase	-
	AFE_3305	hydroxymethylpyrimidine/phosphomethylpyrimidine kinase	-
**Microbial metabolism in diverse environments**	*AFE_0915*	acetyl-CoA carboxylase biotin carboxylase subunit	-
	AFE_0044	TQO small subunit DoxD	-
	AFE_0048	TQO small subunit DoxD	-
	AFE_0423	aconitate hydratase	-
	AFE_0660	malic enzyme-like NAD(P)-binding protein	-
	AFE_0692	FdhF/YdeP family oxidoreductase	-
	AFE_0701	hydrogenase	-
	AFE_0702	nickel-dependent hydrogenase large subunit	-

AFE_0729	glutamate synthase small subunit	-
AFE_0749	pyruvate kinase	-
AFE_0779	ring-opening amidohydrolase	-
AFE_0958	NAD(P)/FAD-dependent oxidoreductase	-
AFE_1665	FAD-linked oxidase C-terminal domain-containing protein	-
AFE_1800	enolase	-
AFE_1893	dihydrolipoyl dehydrogenase	-
AFE_2131	aldehyde dehydrogenase family protein	-
AFE_2550	CoB--CoM heterodisulfide reductase iron-sulfur subunit B family protein	-
AFE_2551	4Fe-4S dicluster domain-containing protein	-
AFE_2553	FAD-dependent oxidoreductase	-
AFE_2554	heterodisulfide reductase-related iron-sulfur binding cluster	-
AFE_3051	form I ribulose bisphosphate carboxylase large subunit	-
AFE_3052	ribulose bisphosphate carboxylase small subunit	-
AFE_3122	NADPH-dependent assimilatory sulfite reductase hemoprotein subunit	-
AFE_3123	phosphoadenylyl-sulfate reductase	-
AFE_3125	GTP-binding protein	-
AFE_3250	phosphoglycerate kinase	-
AFE_3283	hydrogenase small subunit	-
AFE_3286	nickel-dependent hydrogenase large subunit	-
AFE_1962	c-type cytochrome	-
AFE_3124	sulfate adenylyltransferase subunit CysD	-
AFE_1505	serine O-acetyltransferase	-
AFE_2663	4-hydroxy-tetrahydrodipicolinate reductase	-
AFE_1821	succinyldiaminopimelate transaminase	-
AFE_1820	2,3,4,5-tetrahydropyridine-2,6-dicarboxylate N-succinyltransferase	-
AFE_3251	type I glyceraldehyde-3-phosphate dehydrogenase	-
AFE_0696	S-(hydroxymethyl)glutathione synthase	-
AFE_1664	glycolate oxidase subunit GlcE	-
AFE_1663	glycolate oxidase subunit GlcF	-
AFE_3253	class II fructose-bisphosphatase	-
AFE_0730	glutamate synthase large subunit	-
AFE_2222	glutamate--tRNA ligase	-
AFE_1815	2,3-bisphosphoglycerate-independent phosphoglycerate mutase	-
AFE_3300	hydroxymethylbilane synthase	-
AFE_3303	glutamate-1-semialdehyde 2,1-aminomutase	-

	AFE_1521	nitrogenase molybdenum-iron protein alpha chain	-
	AFE_1522	nitrogenase iron protein	-
	AFE_0628	pyridoxamine 5'-phosphate oxidase	-
	AFE_2924	glucose-6-phosphate isomerase	-
	AFE_1816	phosphoenolpyruvate synthase	-
	AFE_3249	pyruvate kinase	-
	AFE_0629	ribose-5-phosphate isomerase RpiA	-
	AFE_0539	sulfate adenylyltransferase	-
	AFE_3252	transketolase	-
	AFE_2633	triose-phosphate isomerase	-
**Nitrotoluened egradation**	*AFE_0702*	nickel-dependent hydrogenase large subunit	-
	AFE_3283	hydrogenase small subunit	-
	AFE_3286	nickel-dependent hydrogenase large subunit	-
	AFE_0701	hydrogenase	-

The symbol "+" indicates an increase in gene methylation levels under copper stress, while the symbol "-" denotes a decrease in gene methylation levels.
